# New Frontiers in Genetics, Gut Microbiota, and Immunity: A Rosetta Stone for the Pathogenesis of Inflammatory Bowel Disease

**DOI:** 10.1155/2017/8201672

**Published:** 2017-08-02

**Authors:** Mingxia Zhou, Jing He, Yujie Shen, Cong Zhang, Jiazheng Wang, Yingwei Chen

**Affiliations:** ^1^Department of Gastroenterology, Xinhua Hospital, School of Medicine, Shanghai Jiao Tong University, Shanghai 200092, China; ^2^Department of General Surgery, Huashan Hospital of Fudan University, Shanghai 200040, China; ^3^Shanghai Institute for Pediatric Research, Shanghai 200092, China; ^4^Shanghai Key Laboratory of Pediatric Gastroenterology and Nutrition, Shanghai 200092, China

## Abstract

Inflammatory bowel disease (IBD), which encompasses ulcerative colitis (UC) and Crohn's disease (CD), is a complicated, uncontrolled, and multifactorial disorder characterized by chronic, relapsing, or progressive inflammatory conditions that may involve the entire gastrointestinal tract. The protracted nature has imposed enormous economic burdens on patients with IBD, and the treatment is far from optimal due to the currently limited comprehension of IBD pathogenesis. In spite of the exact etiology still remaining an enigma, four identified components, including personal genetic susceptibility, external environment, internal gut microbiota, and the host immune response, are responsible for IBD pathogenesis, and compelling evidence has suggested that IBD may be triggered by aberrant and continuing immune responses to gut microbiota in genetically susceptibility individuals. The past decade has witnessed the flourishing of research on genetics, gut microbiota, and immunity in patients with IBD. Therefore, in this review, we will comprehensively exhibit a series of novel findings and update the major advances regarding these three fields. Undoubtedly, these novel findings have opened a new horizon and shed bright light on the causality research of IBD.

## 1. Introduction

Numerous evidence has shown that inflammatory bowel disease (IBD) is emerging as a worldwide epidemic, particularly in developing countries with ongoing industrialization and urbanization [1–3]. It has been well documented that IBD is a multifactorial disease that is mainly manipulated by a series of interactions between genetics, environmental factors, and gut microbiota, as well as immune responses [4–6]. To date, totally, 201 susceptibility loci have been identified for IBD via large scale genomewide association studies (GWAS) and transethnic association studies. This progress paralleled with the advance of next-generation sequencing technologies uncovered the association between rare gene variations and very early-onset IBD (VEO-IBD). Epigenetics, as a new emerging field, also shed bright light on IBD pathogenesis. Furthermore, the genetic risk factors do not act alone but in cooperation with environmental factors, particularly the gut microbiota, to drive the pathogenesis of IBD. Intriguingly, gut microbiota, a field that has continuously sparked numerous researchers' enthusiasm over the past decade, is likely to be the most important environmental factor in IBD pathogenesis. However, we are very bacteria-centric when we look at this term, and only a handful of studies had focused on the viral components (gut virome), fungi or protozoa [7]. Thus, in this review, we will highlight a range of novel findings regarding IBD-specific dysbiosis in bacterial intestinal microbiota, fungal microbiota, and the gut virome, as well as a striking proposal: helminth infection can impact microbial communities and provide protection against intestinal inflammation. Simultaneously, in the gut, the microbiota and immunity undergo constant crosstalk. We will present a simple overview on innate and adaptive immune responses in IBD and then focus on the novel areas inflammasomes, damage-associated molecular patterns (DAMPs), and regulatory RNAs to advance our current understanding of antihomeostatic and prohomeostatic responses in the complicated immunopathogenesis of IBD. Collectively, in this review, we will not only sum up new evolutionary activity in these three fields but also offer insights into future therapeutics.

## 2. Genetics and Epigenetics in IBD

### 2.1. Current Genetic Architecture

The study of IBD genetics has reached an extraordinary milestone over the past decades and has significantly contributed to our understanding of IBD pathogenesis [8]. Particularly in prevailing Caucasian populations 163 susceptibility loci have been identified for IBD basically via the large scale GWAS among approximately 75000 patients and controls. Excitingly, a recent transethnic association study has identified 38 additional new loci for IBD risk with the involvement of more than 20000 individuals [9]. More recently, a deep resequencing of 131 CD associated genes in 500 Korean CD cases and 1000 controls was performed and confirmed 3 previously reported risk loci and 8 novel risk loci [10]. In addition, compelling evidence provided by previous population-based cohort studies indicated that the risk of IBD was increased eight- to tenfold among relatives of UC or CD propositus. Moreover, twin and family studies have suggested that when a child suffered from CD, the risk of developing CD in another sibling had increased 26-fold, compared with a 9-fold increase in UC [11]. Overall, genetic factors contributed greatly to the predisposition of IBD. More interestingly, among a total of 201 loci, 137 conferred risk to both UC and CD (designed as IBD loci), whereas 37 loci were specific to CD and the remaining 27 loci were unique to UC. The majority shared risk loci across divergent populations, mirroring the clinical and pathological similarities observed in both UC and CD.

### 2.2. Biological Implications of 38 Newly Associated IBD Susceptibility Loci

Previous GWAS have given prominence to several critical pathways underlying genetic susceptibility of IBD involving T cell signaling, innate immunity, and epithelial barrier function. To meet the need for fine mapping, here we enlarged the extent of pathways within 38 newly identified susceptibility loci to ascertain causal variants. Previous studies have identified that the genes LAMB1 and HNF4a16 could explain the impaired intestinal barrier function in IBD. Besides, the newly identified OSM could act as a modulator of barrier-protective host responses in intestinal inflammation. What is more, ATG16L1 and IRGM, two pivotal autophage-related genes in host innate immune response in CD, gradually fade away and now the brilliance is covered by ATG4, a novel identified gene in promoting the autophagy process in CD patients. In addition, AHR, CCL20, CD28, LY75, NFATC1, and NFKBIZ, which are newly identified candidate gene members, could regulate T cell responses to a certain extent [9]. Selected meaningful candidate genes in the 38 newly identified IBD susceptibility loci are summarized in Table 1.

### 2.3. Rare Variants and VEO-IBD

Despite most cases being associated with polygenetic contributions toward genetic susceptibility, a series of rare variants has been uncovered through a combination of gene mapping and exome sequencing of specific patients, which are always monogenic and associated with early-onset IBD (EO-IBD) or VEO-IBD, but without the detection spectrum of a GWAS [12, 13]. For instance, a novel variant in CTLA-4 was identified recently to be associated with early-onset CD and autoimmunity [14]. Recent exome sequencing analysis revealed that patients with VEO-IBD carry the rare heterozygous missense variants in IL10RA and novel variants in genes (e.g., MSH5 and CD19) associated with primary immunodeficiency [15]. Moreover, a novel variant in neutrophil cytosolic factor 2 (NCF2) has been found in 4% of patients with VEO-IBD compared with 0.2% of controls, and this variant results in reduced protein binding and partial inhibition of oxidase function [16]. To date, studies of rare variants in IBD genetics supported the link between VEO-IBD and intestinal epithelial barrier defects, Treg cells, neutrophils, T cells, B cells, the IL-10 signaling pathway, and hyperinflammatory and autoinflammatory disorders [12, 17].

### 2.4. IL-10 Signaling Pathway: Master Regulator of Intestinal Mucosal Homeostasis

IL-10 plays a critical role in the regulation of anti-inflammatory responses and the maintenance of intestinal mucosal homeostasis in gastrointestinal tract. Once IL-10 binds its tetrameric receptor complex, it activates tyrosine kinase 2 (Tyk2) and Janus kinase 1 (JAK1), leading to the phosphorylation of signal transducer, the activator of transcription 3 (STAT-3), and the transcription of downstream target genes, finally promoting the expression of anti-inflammatory effectors [18]. Subsequent studies indicated that patients with either IL-10 or IL-10 receptor mutations appeared to have severe colitis and developed severe IBD at an early age [19]. In a recent study, the IL10-related mutation accounted for 38.5 percent of all VEO-IBD in China. However, this figure seems unconvincing due to selection bias and the relative small numbers of cohort studies. Thus, it is imperative to establish countrywide multicenter studies of VEO-IBD to explore the relationship between genotype and phenotype.

### 2.5. IBD Genetics in Asian Populations

Most of our current understanding of IBD genetics is grounded in European population studies, while, increasingly, genetic association studies have begun shedding light on Asian populations. The first GWAS conducted on a Japanese population using single nucleotide polymorphism (SNP) markers identified the first CD-related susceptibility gene, TNFSF15, whereas its associations were pretty modest in European populations. Subsequent studies have shown East Asian CD patients do not harbor ATG16L1 and NOD2 variants, which are specific in European ancestry populations, suggesting that some IBD susceptibility genes are shared across populations, while others are not. These differences between Western and Asian populations in genetic susceptibility to IBD were supported by succeeding studies [20]. More recently, Korean and Japanese GWAS identified ATG16L2 as a novel susceptibility gene for CD, but its genetic risk needs to be validated in larger cohort studies in Asian or in other populations [21, 22]. In contrast, there is a larger overlap ratio between European and Asiatic populations in UC-related susceptibility loci compared with CD [23]. To date, the majority of genotyping studies previously reported from ethnically distinct populations were conducted using SNP chips grounded on Caucasian genetics. We therefore expect to compile SNP chips based on Asiatic populations for further research to explore more about IBD genetics and pathogenesis.

### 2.6. Epigenetics: A Bridge Connecting Genetics and the Environment

**Figure 1:**
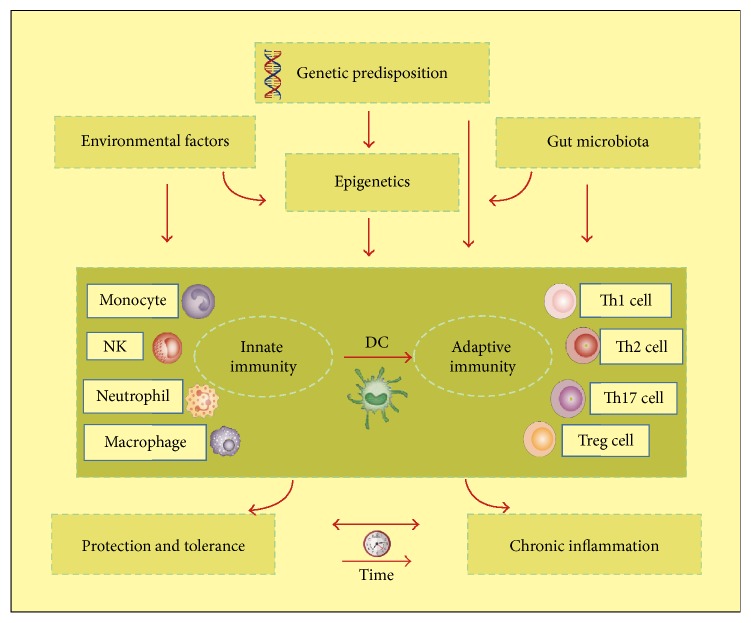
Roles for genetics and epigenetics in IBD pathogenesis. Epigenetics, acting as the mediators of genetic and environmental factors, along with genetic, external environmental factors and internal gut microbiota, participate in motivating the host immune system. The consequence of the following immune response is whether insults tolerance or chronic inflammation initiation and development occur.

However, evidence has suggested that all identified genetic factors only account for a small part of disease variation, that is, 8.2% for UC and 13.1% for CD, respectively, hinting that epigenetic factors, acting as the mediators of the genome and environmental factors, may play significant roles in IBD pathogenesis and affect its development and progression [24, 25]. Therefore, as a new emerging field, the studies of epigenetics provide a novel perspective in IBD pathogenesis. The complex network between genetics, epigenetics, gut microbiota, and immunity is summarized in Figure 1. Emerging studies proved that promoter hypermethylation of many genes (e.g., CDH1, p16, and MDR1) was detected in high frequencies in IBD patients. One IBD epigenome-wide methylation association study identified 51 differentially methylated genes by using whole-blood DNA from patients with IBD and controls, including some involved in immune system activation (IL21R, RPIK3, and MAPK) [26]. Besides, a growing body of evidence demonstrated that the posttranslational modifications of histones, especially acetylation, are correlated with the regulation of inflammation gene expression. There was a significant increase in histone 4 acetylation and lysine residues (K) 8 and 12 especially in the inflamed mucosa of 2,4,6-trinitrobenzene sulfonic acid (TNBS) induced mice colitis. Moreover, dextran sodium sulphate- (DSS-) treated rat models also observed acetylated H4 in inflamed tissues and Peyer's patches. In addition, H4 acetylation was upregulated remarkably in Peyer's patches and inflamed tissues of CD patients [27]. Butyrate, a histone deacetylase inhibitor, formed during fermentation of dietary fibers by the gut microbiota, which participates in the maintenance of intestinal barrier function and a homeostatic reduction in the production of IL-8 by epithelial cells. Butyrate could increase the expression of NOD2 by promoting histone acetylation in its promoter region. And Toll-like receptor 4 (TLR4), a regulator of intestinal homeostasis, could also be regulated by histone deacetylation [28]. All these results have given evidence for a close association between histone acetylation and inflammation and may provide a new therapeutic target for IBD mucosal inflammation.

### 2.7. Future Direction and Outlook

The dramatic expansion of susceptibility loci along with developments in whole genome sequencing and whole exome sequencing techniques have identified considerable genetic targets associated with IBD. The large scale fine mapping approaches, which are extension of current large scale genotyping studies, are expected to narrow down the scope to identify causal variants to promote further functional studies for IBD. Undoubtedly, future functional and expression quantitative trait loci (eQTL) studies will improve our understanding of IBD pathogenesis [29]. Future work needs to determine how these specific variants affect messenger RNA (mRNA) levels and consequently protein levels, which will provide further insight into the mechanisms of IBD pathogenesis. Epigenetics analysis is more likely to be integrated into larger bioanalytical models alongside other emerging IBD research disciplines, such as metagenomics, transcriptomics, metabolomics, glycomics, and glycoproteomics.

## 3. Gut Microbiota: Unraveling the Genetic and Environmental Interactions in IBD

**Figure 2:**
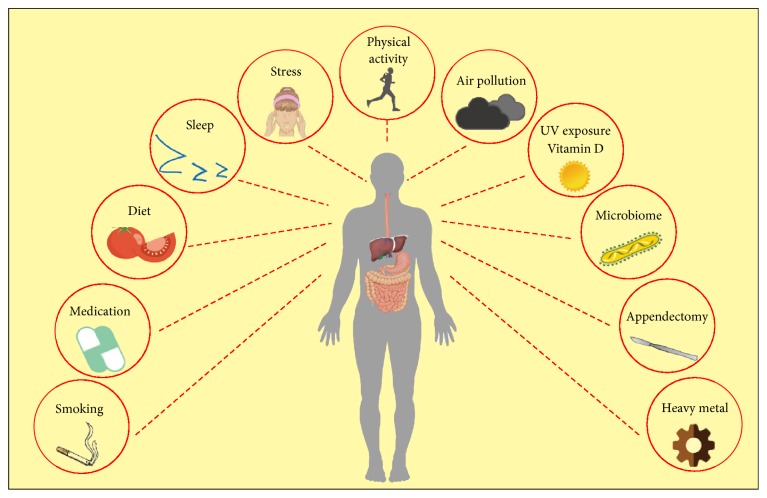
Environmental risk factors involved in IBD pathogenesis. External environmental factors such as air pollution, UV and heavy metal exposure, smoking, and diet, together with some internal environmental factors such as gut microbiota dysbiosis and appendectomy, play significant roles in the development of IBD.

In spite of the genetic landscape remaining considerably stable over the years, the incidence of IBD has shown an increasing trend worldwide, particularly in developing countries with industrialization and urbanization, which are mainly explained by environmental factors via accumulating epidemiological, clinical, and laboratory evidence. For example, twin studies have provided important clues for identifying hereditary and environmental contributions to IBD. Intriguingly, there was a significant increase in the risk and incidence when immigrants moved from countries with low incidence of IBD to countries with high incidence, and the increase was most apparent in the second generation compared with the first generation. What is more, populations in some countries such as Japan, China, and India (previously considered low risk areas) are witnessing a remarkable increase in IBD incidence [30, 31]. Such drastic changes in short time periods are unlikely to be due to genetic factors, which powerfully emphasize the key role of the environment in IBD pathogenesis. Relevant modifying environmental factors throughout our life include diet, smoking, medications, major life stressors, microbiota, vitamin D intake, UV exposure, air pollution, outdoor physical exercises, appendectomy, and impaired sleep [32–37]. The environmental factors involved in IBD pathogenesis are summarized in Figure 2. Most importantly, gut microbiota, a field continuously sparking numerous researchers' enthusiasm in recent years, is likely to be the most important environmental factor in IBD pathogenesis.

### 3.1. Gut Microbiota: An Excellent Environmental Driver in IBD

**Figure 3:**
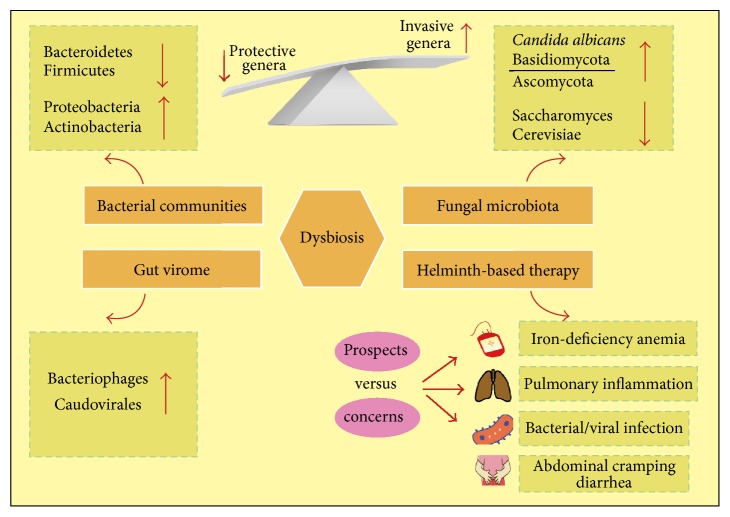
The dysbiosis of gut microbiota on the intestinal mucosal surface is characterized by low richness of protective genera but high richness of invasive genera. The altered profiles and functions of intestinal bacteria, fungi, and viruses under conditions of dysbiosis contribute to IBD pathogenesis. The latest concerns and potential risks about Helminth-based therapy are also summarized in this figure.

The human intestine harbors a complex and vast number of various bacteria, bacteriophages, fungi, and viruses, collectively called “gut microbiota.” We have been very bacteria-centric over the last decades, when we consider this term, and only a handful of studies had focused on the viral components (gut virome), fungi or protozoa. Early studies involving gut microbiota in IBD pathogenesis were centered on identifying a potential criminal bacterium with or without virulence properties that could trigger the inflammatory cascades typical of IBD that led to tissue destruction, such as the proposed* Escherichia coli* [56, 57]. However, current studies have shifted the focus with the realization that altered gut microbiota or dysbiosis as a whole played significant roles in IBD development [7, 58]. More specifically, the abnormal microbial ecosystem is characterized by low species richness but high density colonization and invasion on the mucosal surface. The altered profiles of intestinal bacteria, fungi, and viruses that contribute to IBD pathogenesis are summarized in Figure 3. Overall, IBD involves an aberrant immune response to the gut microbiota in individuals with genetic susceptibility [59]. The imbalance of microbiota communities drives pathogenicity mainly through the restriction of protective bacteria compounds or the expansion of proinflammatory species.

#### 3.1.1. Gut Bacterial Communities: Altered Composition in IBD

It has been estimated that the gut microbiota in a healthy individual contains more than 1000 different species, including approximately 10^14^ bacterial cells, which are often seen as two major phyla: the Firmicutes and Bacteroidetes; the remainder belongs to two rarer phyla, that is, Actinobacteria and Proteobacteria [60–62]. The application of the novel 16S-based next-generation sequencing has provided a cornerstone of microbial composition and the functional genes and taxonomy along with remarkable interindividual diversity. By contrast with healthy gut flora, the dominant phyla Bacteroidetes and Firmicutes are decreased distinctly in IBD patients, whereas Proteobacteria and Actinobacteria are increased considerably [62]. Accumulating studies have indicated that the reduced biodiversity of gut flora is in accordance with the decreasing complexity of the phylum Firmicutes and is also associated with temporal instability of the dominant taxa in IBD [63, 64]. Most remarkably, a number of protective genera such as* Bifidobacterium*,* Faecalibacterium*, and* Lactobacillus*, which have been reported to protect against mucosal inflammation via downregulating inflammatory cytokines and upregulating anti-inflammatory cytokines, are underrepresented in IBD [65–67]. Specifically, changed genera in ileal Crohn's disease (iCD) patients include the expansion of Enterobacteriaceae and decreasing species such as* Faecalibacterium* and* Odoribacter* [68].

#### 3.1.2. Invasive Bacteria Enriched in IBD May Aggravate Disease

In addition, a range of studies has observed an increased load of aggressive bacteria that are attached to the intestinal mucus layer in IBD patients. The* adherent-invasive Escherichia coli* (AIEC) pathovar, for instance, is at higher richness in CD mucosal biopsies compared to healthy controls. While in UC patients, the overall density of attached bacteria is greater than that in healthy subjects. Another group of invasive and adherent bacteria is* Fusobacterium*. The* Fusobacterium varium* has been detected at higher levels in inflamed colonic mucosal biopsy specimens from UC compared with those from control individuals. There is a positive correlation between its invasive ability and host status of IBD, hinting that invasive* Fusobacterium *may affect IBD pathological processes [58, 65, 69]. Collectively, these data clearly identified IBD as associated with the dysbiosis of gut microbial community; however, whether these alternations are the cause or the consequence of IBD remains to be illustrated in further research.

#### 3.1.3. Functional Shifts in IBD

Unlike the remarkable variability of human microbial taxa between individuals, the functional composition of gut bacterial microbiota is dramatically stable. Compared with merely 2% of the genus-level clades, 12% of metabolic pathways differed notably between IBD patients and healthy subjects in a metagenomic study of the IBD microbiome [62, 65, 70]. An integration of metagenomics and metaproteomics studies has indicated that the overall butyrate levels and short chain fatty acids (SCFAs) levels were decreased in iCD patients, which was in line with the decrease of SCFAs-producing Firmicutes phylum observed in taxonomic profiling studies [71, 72]. Another shift is the decreased ability of amino acid biosynthesis, as well as the carbohydrate metabolism, whereas there is an increased ability in nutrient transportation from available inflamed areas and damaged tissues [62, 65]. The IBD metagenome also identified that the biosynthesis and transportation of compounds beneficial to oxidative stress, such as riboflavin and glutathione, are increased in UC patients [62, 65, 70, 73].

#### 3.1.4. Current Understanding and Future Directions

Despite correlative microbial studies having constantly renewed this area, to date our understanding of the dynamic role the gut microbial community plays in IBD pathogenesis remains incomplete. The prevalent probiotic products used as IBD supplementary therapy have modest clinical efficiency, while the emerging fecal microbiota transplantation (FMT) seems radical but promising to recover the disturbed intestinal microbiota homeostasis at least in the short term of IBD [74, 75]. Mechanically, FMT was supposed to increase gut microbiota diversity, provides colonization resistance from invading pathogens, and introduces “healthy” microbes for IBD patients, which are essential for the maintenance of intestinal epithelial integrity, the limitation of gut permeability, and the regulation of systemic and local inflammation [76]. However, no consensus about FMT has been made so far; the mechanisms are still lacking, and its long-term safety and efficacy seems controversial [77–79]. In addition, it is likely that special laboratory-designed bacterial products may soon substitute for FMT to achieve similar success in the treatment of IBD [80, 81]. Therefore, it is imperative now to promote the field of gut microbiota in IBD pathogenesis and therapeutics to achieve greater progress in further research.

### 3.2. Dysbiosis in Fungal Microbiota

Over the last decade, it is quite regrettable that we have overlooked the essential role of fungal microbiota in the pathogenesis of IBD, despite some crucial clues [82–84]. For example, one study in 2012 indicated that two pivotal molecules, Card9 and Dectin1, are engaged in antifungal immunity, and their mutation or deficiency strongly affects susceptibility to gut inflammation and the balance of fungal microbiota [82, 85].

#### 3.2.1. Distinct Altered Composition and Diversity of Fungal Microbiota in IBD

Apart from bacterial dysbiosis, a recent study made by Ray also identified a distinct altered composition and biodiversity of fungal microbiota in IBD patients' feces [86]. Particularly, the composition of fungal microbiota is skewed, with an increased percentage of* Candida albicans*, an increased ratio of Basidiomycota/Ascomycota, and a declined proportion of* Saccharomyces cerevisiae *compared with healthy subjects. One prominent feature in flared IBD is that the decreased Ascomycota was replenished by the increased Basidiomycota; thus this ratio could be an index of fungal dysbiosis [87]. In addition, compared with IBD in a remission stage, the number and proportion of* C. albicans* were increased remarkably when IBD was aggravated. Therefore,* C. albicans* and* S. cerevisiae* may exert disruptive or protective influences on the host inflammatory process, respectively. However, the alterations of fungal diversity were only observed in UC patients. In CD patients, particularly with ileum involvement, the specific gut environment encouraged fungi at the cost of bacteria [86, 88, 89]. Interestingly, this phenomenon may result from the bile acid absorption and antimicrobial peptide production at the ileum.

#### 3.2.2. Intriguing Correlations between Bacterial and Fungal Microbiota

Bacteria and fungi keep a harmonious relationship and coexist in the host gut in normal conditions. Once this balance is interrupted by antibiotics, the primary fungi will expand greatly at the expense of bacteria. The intestinal mycobiota could reduce immune tolerance and lead to inappropriate activation of some pathways designed to protect from pathogen invasion. And accumulating evidence has supported the notion that molecules in fungal cell walls such as chitin, mannans, and *β*-glucans could motivate components of the host innate immune system (e.g., TLR4, TLR2, dectin-1, CD5, and CD36) and the complement system in the pathogenesis of IBD.

### 3.3. Gut Virome: A Novel Frontier in IBD Pathogenesis

The neglected gut virome has been reported to yield many benefits in humans and has been deeply implicated in physiology, immunity and inflammation processes [90, 91]. The development of high-throughput sequencing techniques in recent years has triggered much attention and interest in the altered composition and function of the gut virome in the pathogenesis of chronic inflammation diseases such as IBD. In general, more than 10^9^/g virions can be found in human feces. To a large extent, the human gut virome is comprised of phages while eukaryotic viruses such as DNA or RNA viruses only occupied a small minority [92–94].

#### 3.3.1. IBD-Specific Changes in the Gut Virome

In IBD mucosal biopsies, the numbers of phages are higher and the populations differ from healthy controls, with considerable interindividual diversity and variation [90]. Norman et al. identified five specific subtypes of Caudovirales including* Lactobacillus*,* Lactococcus*,* Clostridium*,* Enterococcus*, and* Streptococcus* being related to IBD pathogenesis. They also observed an expansion in Caudovirales abundance in CD compared with a lesser level in UC samples. Notably, there was a negative correlation between phages richness and bacterial diversity. The composition of intestinal bacterial communities might be, in part, determined by the dynamics of relationships between bacteria and bacteriophage [91]. Specifically, some Caudovirales taxa were negatively correlated with Bacteroidaceae bacterial family in CD, whereas other* Caudovirales *taxa were positively correlated with Pasteurellaceae, Prevotellaceae, and Enterobacteriaceae. These intriguing findings were not seen in UC. Although subsequent validation cohort studies failed to confirm the availability of these data, this research significantly identified the reverse relationship between phage richness and bacterial diversity. However, which one acts as the promoter of the other and what role they play during the initiation of inflammation and its progression need further exploration in the future.

#### 3.3.2. From Dysbiosis to Chronic Inflammation via Four Pathways

The intestinal transition from being normal to being inflammatory may be attributed to phages via the induction of an imbalance between commensal bacteria and pathobionts. de Paepe et al. have postulated four ways by which phages can impact the gut ecosystem. In the first “kill the winner” and the second “biological weapon” models, the phages act as the killers of dominant bacteria or another competitor to shape the gut microbiota ecosystem. In the third “community shuffling” model, temperate phages could lyse their previously lysogenic host upon induction. In the last “new bacterial strains emergence” model, temperate phages could impact the ecosystem via carrying genes modifying bacterial phenotypes instead of killing bacteria [95–99]. Ultimately, whatever pathway it is, the gut virome induces the intestinal dysbiosis and triggers IBD.

### 3.4. Using Helminthes Infection to Ameliorate Gut Inflammation: A Striking Proposal

A recent publication in Science Magazine has linked the gut microbiota with gastrointestinal helminthes and unveiled an intimate crosstalk between them. Harris brought forward a new concept that infection with helminthes can protect from IBD by preventing the expansion of commensal Bacteroidetes in genetically susceptible mice [100]. Among all the genetic susceptible risk factors associated with IBD, the first identified was nucleotide-binding oligomerization domain 2 (NOD2), which has been characteristic of recognizing and responding to bacteria and inducing a program of antimicrobial and inflammatory gene expression. One 2014 study indicated that Nod2^−/−^ mice exhibited a series of inflammatory abnormalities such as goblet cells deficiency and proinflammatory member Bacteroidetes expansion [101]. While a great step forward made by Harris showed that infection with helminthes in Nod2^−/−^ mice can restrict the expansion of Bacteroidetes, restoring goblet cell numbers and epithelial barrier integrity to protect against gut inflammation [100]. Accumulating evidence has supported the notion that some immunoregulatory capacity of parasites can modulate intestinal microbial communities in a direct fashion or indirectly. More specifically, a significant increase in protective Clostridiales species was observed in helminthes-infected Nod2^−/−^ mice, which in turn limited the colonization of Bacteroidetes [102]. Interestingly, results in the human population mirrored a similar phenomenon in that people residing in rural areas have higher numbers of Clostridiales species and microbial biodiversity but a lower quantity of Bacteroidetes in comparison with urban areas [103].

#### 3.4.1. Helminthes-Based Therapy: Prospects and Concerns

Epidemiological and experimental data to date support the idea that eradicating helminth's infections in well-developed countries underlies the increasing incidence rate of autoimmune and inflammatory diseases [104]. Therefore, the proposal of altering our gastrointestinal microbial environment by exposure to specific bacteria deliberately (in particular, fecal transplants and probiotics) or parasite-derived therapy to ameliorate intestinal inflammation has gained considerable momentum in recent years [104]. Simultaneously, helminths therapy has raised some safety concerns due to it differing from natural infection and exposure. Although clinical trials conducted to date in IBD patients have revealed no side-effects, concern has been voiced that parasites may travel to other organs, such as the lungs, and cause pulmonary inflammation, may trigger iron-deficiency anemia in malnourished children, may cause abdominal cramping and diarrhea, may promote AIDS progression in HIV patients, and may predispose patients to bacterial or viral infections [105–107] (Figure 3). Compared with a recent report that the alternations of gut microbiota induced by helminths can suppress inflammation in the lung, these results underline the crucial impacts that helminths and gut microbiota have on human diseases and open a novel perspective on IBD pathogenesis [102, 108].

### 3.5. Current Questions and Future Directions

To date, our understanding of the dynamic role of gut microbiota in IBD patients still remains shallow. There is still a long way to go and some crucial questions remain to be answered regarding the contributions of gut microbiota to IBD pathogenesis. Can the gut microbiota modulate the host immune system in a disease-specific manner? How do bacteria and fungi interact with each other to influence the development of IBD? And whether the host immune system could be affected directly or indirectly by the fungal-fungal interactions? Are the interactions species specific? In addition, the outstanding biological questions of interest enabled by future prospective and longitudinal studies would be as follows: (1) to identify the specific role of gut microbiota in triggering IBD pathogenesis; (2) to determine whether microbial composition predicts subsequent risk of activity flares; and (3) to predict the response to pharmacotherapeutics through the assessment of gut flora.

## 4. The Interplay between Gut Microbiota, Epithelial Barrier, and Immunity

In the gut, the microbiota and immunity undergo constant interactions. Gut microbial colonization in mucosal tissues during infancy has a profound influence on regulating the host mammalian immune system development and maturation. More recently, a study nicely demonstrated that such influences by gut microbiota may be more evident during pregnancy, during which the number of specific immune cells and their activity in offspring were regulated by gut microbiota colonization [109]. All of that is aimed at establishing a balanced relationship between tolerance and protective immunity. The receptors of innate immunity such as Toll-like receptors (TLRs) can sense a wide range of signals such as microbe-associated molecular patterns (MAMPs) to regulate and maintain intestinal homeostasis. However, both gut microbiota and immunity contribute great and equal importance to intestinal homeostasis. On the one hand, the products of immune cells (e.g., defensins, mucus, and IgA) dominate the intestinal microbial ecosystem [110]. Conversely, mucosal immunity can be regulated by gut microbiota [111]. One study made by Elinav et al. in 2011 indicated that NLRP6 inflammasome deficient mice were characterized by inflammatory cell recruitment and exacerbation of intestinal colitis induced by exposure to DSS. And the deficiency of NLRP6 in mouse IECs results in altered fecal microbiota characterized by expanding bacterial phylum Bacteroidetes [112]. In addition, Levy et al. had identified distinct microbiome-modulated metabolites as in vitro and in vivo regulators of NLRP6 inflammasome activation, epithelial IL-18-induced antimicrobial peptide secretion, and downstream control of microbiota [113].

### 4.1. Epithelial Barrier: An Interface for the Crosstalk between Gut Microbiota and Immune System

The interactions among intestinal epithelial barrier, commensal bacteria, and mucosal immune cells provide the platform for establishing the intestinal dynamic and flexible environments that involve the formation and maintenance of mucosal homeostatic balance between quiescent and active immunity. More specifically, intestinal epithelial cells (IECs), which physically and biologically located between mucosal immune system and the commensal flora, may play a central role in the pathogenesis of IBD, owing to their interaction with the inside host immune system and the outside gut microbiota, respectively.

IECs construct a crucial mucosal defensive barrier system against various bacteria. The temporary disruption of epithelial barrier function results in the invasion of commensal bacteria and the recruitment and activation of proinflammatory immune cells for initiating acute intestinal inflammation. However, the permanent disruption of epithelial barrier function, whether caused by genetic abnormalities or continuous commensal stimulation, eventually leads to the emergence of chronic intestinal inflammation.

According to the current literature, high frequency of epithelial apoptosis has been observed in colon tissues of patients with CD [114]. Accumulating evidence suggests that this aberrant epithelial apoptosis and intestinal inflammation could be the consequences of abnormal penetration of intestinal commensal bacteria through the breakage of epithelial barrier via speeding up IECs apoptosis and subsequent mucosal immune cells' hyperactivation to produce pathological inflammatory conditions.

ATG16L1 is one of susceptibility genes for CD and has been reported to participate in autophagy process [115]. The mice containing hypomorphic ATG16L1 (ATG16L1_HM_) are susceptible to DSS-induced colitis when coinfected with murine norovirus. And mice with epithelial-specific deletion of autophagy-related protein such as ATG5 and ATG7 also show similar abnormal phenotype like ATG16L1_HM_ mice [116]. Interestingly, the elevated inflammatory states of mice could be rescued by antibiotics treatments, suggesting the involvement of intestinal commensal bacteria. Therefore, autophagy process could prevent the development of IBD via the establishment of IECs homeostasis.

When the IECs' secretory molecules such as *α*-defensins are transported into endoplasmic reticulum (ER), the cells will be stressed for the accumulation of misfolded proteins. In order to maintain the homeostasis of ER environment, a serious of transcription factors and enzymes cooperatively engage in the process of unfolded protein responses (UPRs) [117]. The aberrance of UPRs affects the cellular function and eventually leads to the initiation of apoptosis [118]. ER stress mediated by X-box-binding protein 1 (XBP1) plays a key role in the homeostasis of IECs. The hypomorphic variants of XBP1 in humans are important risk factors for IBD. And XBP1-deficiency mice are more sensitive to DSS-induced colitis and could develop enteritis spontaneously [119].

Overall, the aberrance of some biological processes such as apoptosis, autophagy, and the handling of endoplasmic reticulum stress will break the physiological homeostasis of IECs and lead to the pathogenesis of IBD.

### 4.2. An Overview of Innate and Adaptive Immune Responses in IBD

It is a well-established fact that both innate and adaptive immune responses contribute greatly to IBD pathogenesis, together with the genetic factors described earlier. Innate immune responses provide the host with immediate protection to defend against any forms of aggression at the forefront, other than the adaptive immune response. Innate immune response is semispecific, interim, and crucial for preventing microbe invasion, maintaining homeostasis, and contributing to the activation and regulation of the adaptive immune response. The innate immune system is composed of physical and chemical barriers, a variety of immune cells such as macrophages, dendritic cells (DCs), neutrophils, and monocytes. These immune cells act together to trigger inflammation via producing cytokines, chemokines, and antimicrobial agents, which results in phagocytosis of target cells and antigen presentation, along with initiating the adaptive immune system [120]. Particularly, DCs are key players in innate and adaptive immunity' crosstalk and are responsible for T cell activation and eliciting an adaptive immune response. However, the adaptive immune system is a highly specialized system and provides long-lasting immunity (memory). T cells play a pivotal role in the adaptive immune response [121]. The activated naïve T cells (Th0) can differentiate into Th1, Th2, or Th17 cells. Accumulating evidence has suggested that an adaptive immune response contributes greatly to IBD pathogenesis in recent years. More specifically, Th1 responses act as the main drivers of CD, whereas Th2 responses have been thought to drive UC pathogenesis. Recent progress also elaborated that Th17 cells are prominent contributors to IBD pathogenesis [122, 123].

Innate lymphoid cells (ILCs) were initially reported to mediate colitis in mice models of IBD, especially group 3 ILCs, associated with high secretion of Th17 like cytokines such as IL-17 and IL-22. Subsequent studies have identified a similar phenomenon in the mucosa of patients with IBD [122]. According to the current literature, ILCs are surely present in the gut of IBD patients, but the functional data are merely limited to animal studies, whereas several IBD-related genes are expressed and associated with ILC function, hinting that they may be a possible player in IBD pathogenesis [111]. Macrophage-derived IL-12 can trigger aberrant Th1 immune responses to cause intestinal inflammation in patients with CD, while macrophage-derived IL-12 is certainly highly and preferentially expressed in CD patients. It has also been observed that activated T cells from the mucosa of CD patients produce more IFN-*γ* than those from UC and healthy controls [122]. Further studies demonstrated, in UC patients, that IL-5 is produced in remarkably higher amounts. For a long time, CD was thought to be mediated by Th1 responses, while UC was believed to be a Th2 mediated disease, until some evidence suggested that the Th1/Th2 paradigm in CD and UC should be reconsidered. For example, both CD and UC biopsies showed considerable amounts of IFN-*γ*, and there were no significant differences in IL-13 levels from intestinal biopsy' supernatants between UC, CD, and controls [124]. What is more, IL-23-induced Th17 cells act as a key mediator in intestinal mucosa inflammation. However, experimental studies have suggested a confusing role for Th-17 in IBD pathogenesis, by either inducing mucosal inflammation and damage or protecting against inflammation [125]. Th-17 cells are featured by the production of abundant cytokines, such as IL-17A, IL-17F, IL-21, and IL-22. Taking the DSS-induced colitis mice models, for example, IL-17 knockout mice exhibited more severe colitis, whereas IL-17F deficiency mice showed moderated colitis, which suggests completely adverse functions between Il-17A and IL-17F.

### 4.3. Novel Focus of IBD Immunopathogenesis

The collected data so far indicates that all chronic diseases, including IBD, are not merely sophisticated but also coincide with disorders of intestinal mucosa homeostasis. Recent progress linking the emerging novel areas of inflammasomes, DAMPs, and regulatory RNAs to intestinal inflammation is elaborated here to provide a new understanding of antihomeostatic and prohomeostatic responses and the complex immunopathogenesis in IBD.

#### 4.3.1. Role of Inflammasomes in Regulating Intestinal Mucosal Immune Response

**Figure 4:**
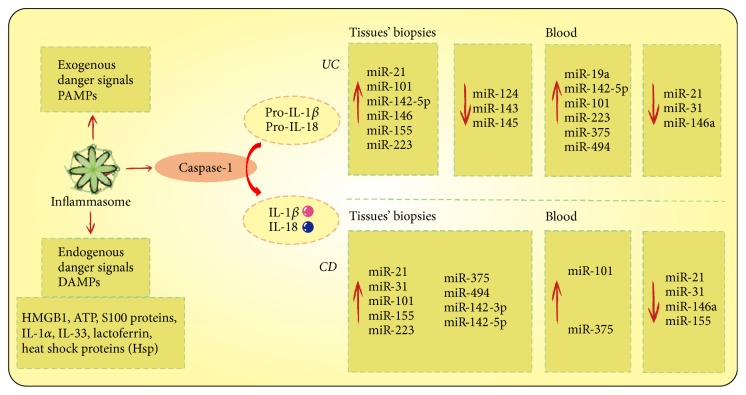
Inflammasomes recognize various exogenous danger signals (PAMPs) and endogenous danger signals (DAMPs) and respond by activating caspase-1 and promoting the production of IL-1*β* and IL-18. The dysregulated expression of tissue and blood miRNAs and insufficient miRNA-mediated suppression could lead to an excessive immune response and inflammation, and these differential expression miRNAs may help distinguish between UC and CD and provide potential targets for early detection and therapy.

Inflammasomes are cytosolic multiprotein complexes that serve as signaling platforms to sense and recognize a large array of exogenous stimuli, microbial products, and a number of damage and stress related DAMPs, which normally indicate the disruption of cellular homeostasis [126]. An inflammasome complex contains a sensor protein, an adaptor protein, and a zymogen procaspase-1. Sensor protein can be formed by either members of the Nod-like receptor family (NLRP1, NLRP2, NLRP3, NLRC4, NLRP6, NLRP7, etc.) or the PYHIN family members AIM2 and IFI16 [127]. Once formed, it will trigger the proteolysis of caspase-1 and subsequently lead to an effective inflammatory response via IL-1 family cytokines maturation and secretion of IL-1*β* and IL-18 and simultaneously induce a distinct inflammatory modality of programmed cell death termed pyroptosis. This process is summarized in Figure 4.

*Involving Inflammasomes in Intestinal Homeostasis between the Host and Gut Microbiota.* Recent evidence has highlighted the role of inflammasomes in the orchestration of intestinal innate immune responses and the maintenance of intestinal homeostatic biological functions. What makes the inflammasomes central and specifically attractive in the IBD immunopathogenesis is their significance in the complex crosstalk between the host mucosal immune system and the environment, particularly the microbiota [128]. For instance, NLRP6 controls the intestinal microbial composition and biogeographical distribution. In NLRP6-deficient mice, the aberration in microbial ecology is characterized by the outcomes of Prevotellaceae, pointing to the fact that there is highly improved abundance in fecal microbiota. In addition, the commensal microorganism accumulation, such as Prevotellaceae, was observed in colonic crypts [129].

*The Contentious Role of Inflammasome in IBD: Friends or Foes? *However, a substantial number of studies that have accumulated suggest a dual role for inflammasomes in inflammatory pathways and a confusing role in IBD pathogenesis [130]. NLRP3 was the first reported inflammasome to protect against microbion-driven colitis, and NLRP3-deficient mice had increased susceptibility to experimental colitis, whereas in another study, mice lacking NLRP3 exhibited alleviative colitis severity [111]. These opposite results can more likely be attributed to the composition variation of gut microbiota. As such, the intestinal epithelial cell activation induced by NLRP6 prevented chemically induced colitis through regulating the microbial imbalances. Evidence derived from NLRP6 deficiency-driven dysbiosis sparked epithelial reprogramming, increased chemokine CCL5 production, and predisposed the host gut to low-grade inflammation. Nevertheless, a sharp contrast to its protective role in the gastrointestinal tract is its detrimental effects during gram-positive or gram-negative microbe infections (like* Salmonella typhimurium* and* E. coli*) [131].

#### 4.3.2. Gut Mucosal DAMPs in IBD: Alerting the Host and Promoting Inflammation

Here, we focus on the comparatively underexplored but potentially pivotal contribution to the initiation, maintenance, perpetuation, and amplification of abnormal mucosal inflammation in IBD patients—DAMPs [132].

The tissue damage, along with diverse cell death (apoptosis, necroptosis, necrosis, and pyroptosis) caused by chronic inflammation, is always accompanied by the release of intrinsic proinflammatory materials termed DAMPs. Independently of various PAMPs, DAMPs alert the host immune system to danger signals by inducing the so-called sterile inflammation [133]. Putative DAMPs contain HMGB1, heat shock proteins (HSPs), S100 proteins, ATP, IL-1, IL-33, lactoferrin, and extracellular matrix (ECM) fragments such as hyaluronan, biglycan, fibronectin, and tenascins-C [132].

The innate immune system can precisely and rapidly make a distinction between nonself and self by binding PAMPs/MAMPs via a system named germline-encoded pattern recognition receptors (PRRs). The main types of PRRs include Toll-like receptors (TLRs), NOD-like receptors (NLRs) such as NOD-1 and NOD-2, and RIG-1-like receptors (RLRs). PRRs sense infectious danger signals via discerning evolutionarily relative conserved PAMPs on pathogens or sterile inflammation via recognizing DAMPs [134]. The activation of PRRs eventually converges on NF-*κ*B activation and translocation, resulting in a series of intracellular signaling cascades, promoting the transcription of genes encoding proinflammatory cytokines and chemokines (e.g., IL-1*α*, IL-1*β*, IL-6, and CCL-2), and inflammatory cell recruitment such as neutrophils. Although it is initially beneficial and protective, the persistent release of DAMP will lead to detrimental “collateral damage” and thus play a central role in disease pathogenesis [135].

*DAMP-Mediated Mechanisms in IBD. *A volume of studies has indicated DAMPs (e.g., HMGB1, S100A calgranulin, IL-1*α*, and IL-33) have been found in abundance in active IBD patients, suggesting the persistently and chronic inflamed intestinal mucosa may symbolize abundant DAMP resources [136]. One specific example is activation of RAGE, a multiligand receptor binding HMGB1, S100 proteins, amyloid peptide, and advanced glycation end products. Däbritz et al. suggested RAGE expression is upregulated in inflamed CD intestinal tissues where its ligands are abundant [137]. Several studies have shown RAGE activated by HMGB1 may play a dominant role in neutrophil recruitment and migration, and anti-RAGE antibodies suppress migratory activity and cytokine release in IECs. In addition, the extracellular ATP released by stressed cells can trigger intestinal inflammation through ligation with the purinoreceptor P2X7 receptor, whose expression is upregulated in the mucosa of active CD [138]. And in animal studies the alarmin IL-33 could promote the generation of Treg cells in the inflamed intestine. The alarmin IL-1*α* released by the damaged IECs could reactivate inflammation in mice during clinical remission stage from DSS colitis [136]. Therefore, any substance, infection, or drug injuring the intestinal epithelium could trigger a flare-up of IBD and recurrence of disease through a DAMP-mediated mechanism.

#### 4.3.3. Regulatory RNAs: New Players in IBD

MicroRNAs (miRNAs) are small, endogenous, single-stranded noncoding RNAs, 18–23 nucleotides long, which regulate gene expression at a posttranslational level or influence the process of transcription, eventually causing gene silencing via specific target mRNA degradation and translational inhibition [139, 140]. Recent evidence has indicated that microRNAs, along with other types of noncoding RNAs, such as intriguing long noncoding RNAs (lncRNAs) and circular RNAs (circRNAs), exert considerable regulatory function and are strongly implicated in the pathogenesis of a diverse range of common diseases, including IBD, which provide a new way to improve the comprehension of IBD [141]. Recent evidence suggested a serious of circRNAs functioning as miRNA sponges to regulate gene transcriptional processes and play an important role during the onset and development of many diseases including IBD [142]. The dysregulation of or insufficient miRNA-mediated suppression would result in excessive immune responses and inflammation, both of which are relevant to IBD pathogenesis.

*Expression Differences Help Discriminate between CD and UC. *Circulating RNAs are expressed at different levels in the peripheral blood of IBD patient samples compared to healthy controls. Simultaneously, in the colon tissue biopsies from UC and CD patients, expressions of miRNAs are also changed meaningfully. The differential expression of miRNAs in tissue and blood of IBD patients id summarized clearly in Figure 4. Thus, it is helpful to use differently expressed miRNAs to distinguish between UC and CD diagnostically. The results provide feasibility to use blood as a diagnostic tool and a potential method for IBD early detection.

Several studies support the notion that miRNAs act as crucial regulators of inflammatory responses in intestinal epithelial cells of mammals via NF-*κ*B and IL-6/STAT3 pathways [143]. The expression of miR-124 was downregulated in colon tissues of pediatric patients with active UC, whereas levels of directed targeted phosphorylated STAT3 and its regulative genes such as VEGF, BCL2, and MMP9 were increased, which could accelerate inflammation and promote the pathogenesis of UC in children [144]. Furthermore, miR-143 and miR-145 were downregulated significantly in chronic UC compared to normal colonic mucosa, so we speculated that the loss of miR-143 and miR-145 predisposed the host to chronic inflammation and increased the risk of neoplasm in IBD [145]. What is more, four miRNAs, including miR-192, miR-194, miR-200b, and miR-375, which were previously shown to be enriched in IECs, were declined remarkably in UC while only miR-21 and miR-375 were changed meaningfully in CD patients, with identification where miR-192 does not directly target NOD-2 [146].

Accumulating evidence has suggested that the levels of lncRNA could affect TLR4-driven expression of inflammatory genes and influence the expression of various pro- and anti-inflammatory cytokines. In addition, a number of previously identified lncRNAs, such as lncRNA-Nfkb2 and lncRNA-Rel, coordinated the regulation of its neighboring protein-coding immune response genes, which played a crucial role in immune responses [147, 148]. Thus, targeted management of regulatory miRNAs expression may offer a new way to interfere with inflammatory diseases.

#### 4.3.4. Current Questions and Future Directions

The last decade witnessed a wealth of information about inflammasomes, DAMPs, and regulatory RNAs, leading to a better understanding of their roles in IBD pathogenesis. Despite great progress in elaborating the role of inflammasomes in experimental animal models, few studies have used cells/tissues from healthy subjects and patients with IBD. So far, only a few inflammasome SNPs have been related to CD and none have been associated with UC [149]. Therefore, future researchers should aim to convert the experimental results into human models to discover a more accurate understanding of these intricate conditions. Currently, a number of doubts about DAMP-mediated inflammation in IBD still exist, including (1) deciphering DAMP-PRR and DAMP-DAMP interactions; (2) understanding specific triggers for DAMP release; (3) understanding intricate IBD-specific DAMP biology with their diverse competing effects; and (4) how DAMP-mediated signaling varies depending on context. Moreover, the abnormal miRNAs found in tissues and peripheral circulations were thought to be promising biomarkers for IBD diagnosis and clinical activity. However, considerable difficulties still lie ahead before these optimistic hopes become reality: regulatory miRNAs exist in the thousands and show complicated regulatory mechanisms, and the numbers of dysregulated miRNAs in the tissues and blood of IBD patients are already over one hundred.

## 5. Conclusions

The dramatic expansion of susceptibility loci, along with the advances of next-generation sequencing technologies, has assisted with the identification of considerable genetic targets associated with IBD. Future studies are expected to narrow down the scope to identify causative variants. The epigenetic factors, which act as the mediators of genetic and environmental factors, will have a bright future in the pathogenesis of IBD. Recent progress in gut microbiota has clarified its key importance in IBD pathogenesis. Undoubtedly, several overlooked fields, such as fungal microbiota, enteric virome, and helminthes, have also become very important and potentially groundbreaking research areas, which should be explored in great depth in further studies. Special laboratory-designed bacterial products may soon substitute for FMT to achieve similar effects. By assessing the enteric virome in patients with IBD, we may make a clearer distinction between UC and CD. In addition, although the recent proposal of helminths-derived therapy has gained considerable momentum, the potential risk of serious complications must be considered carefully. Moreover, by better understanding the immune mechanisms in IBD, we are more likely to further advance IBD etiologic studies. Ultimately, with the goal of improving the outcome of patients with IBD, it is imperative now to accelerate the speed of clinical translation applications.

## Figures and Tables

**Table 1:** Selected candidate genes in the 38 newly associated IBD susceptibility loci.

Gene name	Aliases	GO annotations	Previous associated diseases	Novel biological significance in IBD	References
PTGS2	Prostaglandin-endoperoxide synthase 2	Protein homodimerization activity and lipid binding	Colorectal adenoma and peptic ulcer disease	Inhibiting T cell activation and promoting regulatory T cell development	[38–40]

LY75	Lymphocyte antigen 75	Receptor activity and carbohydrate binding	Hodgkin lymphoma and lipid pneumonia	Participating in T cell function and homeostasis	[41, 42]

CD28	CD28 molecule	Identical protein binding and SH3/SH2 adaptor activity	Sezary's disease and mycosis fungoides	A key costimulatory molecule in T cell activation and preventing aberrant immunological responses to intestinal antigens	[43, 44]

CCL20	C-C motif chemokine ligand 20	Cytokine activity and chemokine activity	Rheumatoid arthritis and bejel	Regulating the migration of T cells (especially regulatory T cells) and dendritic cells to the gut	[45–47]

NFKBIZ	NFKB inhibitor zeta	Transcription cofactor activity	Myxoid liposarcoma and brain glioblastoma multiforme	Activate natural killer cell, recruiting monocyte, and regulating Th17 development with ROR nuclear receptors	[48, 49]

OSMR	Oncostatin M receptor	Growth factor binding and oncostatin M receptor activity	Amyloidosis, primary localized cutaneous, 1 and amyloidosis	Promoting intestinal epithelial cell proliferation and wound healing	[50]

AHR	Aryl hydrocarbon receptor	Transcription factor activity, sequence-specific DNA binding and protein heterodimerization activity	Eosinophilic fasciitis and seborrheic dermatitis	Maintaining intraepithelial lymphocyte homeostasis and controlling intestinal microbial load and composition	[51, 52]

PTK2B	Protein tyrosine kinase 2 beta	Transferase activity, transferring phosphorus-containing groups and protein tyrosine kinase activity	Transient cerebral ischemia and osteoporosis	Monocyte migration and neutrophil degranulation	[53]

NFATC1	Nuclear factor of activated T cells 1	Transcription factor activity, sequence-specific DNA binding, and transcription regulatory region DNA binding	Bone epithelioid hemangioma and ventricular septal defect	Supporting lymphocyte proliferation and inhibiting activation-induced cell death	[54, 55]
